# A Supervised Link Prediction Method Using Optimized Vertex Collocation Profile

**DOI:** 10.3390/e24101465

**Published:** 2022-10-14

**Authors:** Peng Wang, Chenxiao Wu, Teng Huang, Yizhang Chen

**Affiliations:** 1 School of Computer Science and Engineering, Southeast University, Nanjing 211189, China; 2 School of Cyber Science and Engineering, Southeast University, Nanjing 211189, China; 3 Chien-Shiung Wu College, Southeast University, Nanjing 211189, China

**Keywords:** link prediction, social network, topological structure, optimized vertex collocation profile, community detection

## Abstract

Classical link prediction methods mainly utilize vertex information and topological structure to predict missing links in networks. However, accessing vertex information in real-world networks, such as social networks, is still challenging. Moreover, link prediction methods based on topological structure are usually heuristic, and mainly consider common neighbors, vertex degrees and paths, which cannot fully represent the topology context. In recent years, network embedding models have shown efficiency for link prediction, but they lack interpretability. To address these issues, this paper proposes a novel link prediction method based on an optimized vertex collocation profile (OVCP). First, the 7-subgraph topology was proposed to represent the topology context of vertexes. Second, any 7-subgraph can be converted into a unique address by OVCP, and then we obtained the interpretable feature vectors of vertexes. Third, the classification model with OVCP features was used to predict links, and the overlapping community detection algorithm was employed to divide a network into multiple small communities, which can greatly reduce the complexity of our method. Experimental results demonstrate that the proposed method can achieve a promising performance compared with traditional link prediction methods, and has better interpretability than network-embedding-based methods.

## 1. Introduction

Link prediction focuses on the interactions between users in social networks. Namely, with the seen network vertexes and network structure information, link prediction aims to predict the likelihood that two vertexes will be linked, if they had not been connected before [1,2,3,4]. The links to be predicted include two types: unseen links and future links. Unseen links actually exist in the network but have not been detected, while future links represent ones that do not currently exist in the network but are likely to exist in the future [1,2].

Vertex information is useful in link prediction. If we can obtain the attributes of a vertex in a social network, we can use such information to calculate the similarities between the vertex and other vertexes to predict the links. However, it is difficult to obtain vertex information in real-world scenarios. For example, some attributes of a user in a social network are usually confidential. Moreover, even if the attribute information of vertexes is obtained, it is difficult to ensure the credibility and integrity of the information [5,6]. Due to the limitations of the link prediction methods based on vertex attributes, link prediction methods based on topology have attracted more and more attentions [1,2]. Different from the attribute information of vertexes that may be confidential or unreliable, the topology information of networks can be directly obtained from the relationships between vertexes [3]. At the same time, link prediction methods based on network topology are general. If we predict links using vertex information, we need to deal with different vertex information for different networks. However, the topology information in different networks is similar, so the link prediction methods using topology as features can be universal. It means that the link prediction methods based on topology structure are robust [7,8].

The link prediction methods based on topological structure employ the topology features to rank the vertex pairs without links [9]. However, implementing an efficient link prediction method based on topological structure is a nontrivial task. First, there is inevitably noise in real-world networks [10]. Second, traditional link prediction methods based on topological structure, such as the Katz method [11], have high time complexity and require pairwise judgments of vertexes in networks, resulting in time-consuming computation. Third, most link prediction methods do not consider the dynamic and temporal evolution in networks. Finally, even weight information of edges is meaningful, it is difficult to assign weights for edges [12,13].

Various topology-based methods are proposed to alleviate the above problems. Through the analysis of the topology, not only can the local structure information of the vertexes be obtained, and then the environmental properties can be inferred, but also the structural information of the network can be obtained, and the structure and characteristics of the network can be analyzed. Those methods use common neighbors, paths and random walk and they do not take full advantage of topology information in the network. Moreover, in recent years, network embedding methods based on deep learning have been proposed, such as LINE [14] and node2vec [15]. However, such methods not only require a lot of model training, but also lack interpretability for the results of link prediction [16]. Vertex collocation profile (VCP), which combines the topological structure methods with the supervised learning methods [17,18], is an efficient way to address the link prediction problem. However, VCP still suffer from the limitations of high computation complexity and many meaningless subgraphs.

To address the above issues and to obtain better topological representation and better interpretability, this paper proposes a supervised link prediction method using optimized vertex collocation profile (OVCP). On the basis of VCP, OVCP first removes redundant subgraphs to reduce the number of subgraphs and decrease the complexity. Then we propose the 7-subgraph to overcome the drawback that the VCP method can only calculate the number of vertexes in subgraphs less than or equal to 5. Meanwhile, 7-subgraph can ensure that it contains more topological information around the vertexes. OVCP only uses the subgraphs with paths between vertexes as features. Finally, the number of these subgraphs is counted and the corresponding feature vectors are obtained for supervised machine learning models to predict links.

In summary, the main contributions of this paper are as follows:

- We propose a method, OVCP, to represent topological context between vertexes in networks. We consider paths as topological structure. According to the “three degrees of influence rule” [19] and the “six degrees of separation” [20], we assume that two vertexes will not connect to each other if the path length between them is longer than 6. Therefore, we ignore the paths longer than 6. For a path between $v_{x}$ and $v_{y}$, we complete the links between any two vertexes on the path. Therefore, we can obtain a topology context with more information. The length of the path in any topological structure is shorter than or equal to 6, and we can store it by a graph with seven vertexes, which is called 7-subgraph;
- We propose a supervised link prediction method based on OVCP. OVCP provides a way to convert a 7-subgraph to a unique address. For a 7-subgraph, we can calculate its address and treat it as a feature. We set a feature vector for each vertex pair $v_{x}$ and $v_{y}$. The value of a feature represents the number of occurrences of a 7-subgraph on the path. If two vertexes connect to each other in a time interval, we will set the vector of them as a true sample. Otherwise, we will set it as a false sample. Then, we use the vectors and their labels to train the supervised learning model for link prediction;
- We propose a vertex pairs selection method based on overlapping community detection algorithm [21]. The complexity of finding vertex pairs that will be likely to link to each other is $O(n^{2})$. In this paper, we use fast unfolding community detection algorithm [22,23] and overlapping community detection algorithm to split the original networks. These two algorithms can split the network into several small communities. We assume that only the vertexes in the same community will connect to each other, which can reduce the complexity. However, the fast unfolding community detection algorithm will ignore some possible links between vertexes in different communities, but the overlapping community detection algorithm will not;
- The experimental results on the DBLP dataset, Facebook Friendship dataset and Facebook Wall dataset demonstrate that our method achieves excellent link prediction performance, which outperforms the traditional link prediction methods based on topology.

The rest of the paper is organized as follows. Section 2 analyzes the related work. Section 3 defines the link prediction problem. Section 4 presents the VCP and OVCP in detail. Section 5 describes the community detection algorithm used by this paper. Section 6 describes the supervised learning methods used by our link prediction model. Section 7 reports the experimental results and Section 8 concludes the paper.

## 2. Related Work

Recently, many link prediction methods have been proposed. These methods can be divided into four categories: (1) Link prediction methods based on vertex attributes; (2) Link prediction methods based on topological structure; (3) Link prediction methods based on learning; (4) Link prediction methods based on social theory.

### 2.1. Link Prediction Methods Based on Vertex Attributes

A simple assumption of these methods is that the more similar two vertexes are, the more likely there is a link between them. We rank the vertex pairs according to their similarity of attributes, and assume that the vertex pairs in the top-k rank would generate links in the future. There are many ways to describe the similarity of vertexes. One of the simplest ways is to use the attributes of vertexes. Anderson et al. used the coincidence degree of user interests to calculate similarity [24]. In addition, Bhattacharyya et al. defined a multi-classification tree model to learn keywords in user profiles and defined similarity between users based on the distance between keywords [25]. Akcora et al. found that the profile information of most users was missing. Therefore, they proposed a link prediction method to infer users profile information according to mutual friends and majority voting, which improved the prediction performance of multi-classification tree model [26].

### 2.2. Link Prediction Methods Based on Topological Structure

The link prediction methods based on topology structure refer to use the topology structure information shared by two vertexes to calculate the similarity between them. common neighbors, paths and random walk are three popular ways to describe the topological structures.

#### 2.2.1. Methods Based on Common Neighbors

These methods mainly use the information of common neighbors to calculate the similarity between two vertexes. CN index (common neighbors) is one of the simplest indexes based on the such assumption that if two vertexes have more common neighbors, they prefer to be linked with each other [12]. Considering the influence of vertex degrees based on common neighbors, many similarity indexes are proposed, such as the Salton index, the Jaccard index, the Sorenson index, the HPI index (hub promoted index), the HDI index (hub depressed index) and the LHN-I index (leicht-holme-newman index). In addition, Adamic and Adar proposed the famous AA index [27] to denote the contribution of common neighbors with less degree was greater than the common neighbors with greater degree. Therefore, it should assign a weight to each vertex according to the degree of common neighbor ($\frac{1}{log(k)}$), where *k* is vertex degree. Inspired by the network resource allocation process, Zhou et al. proposed the RA index (resource allocation) [28]. RA has a similar effect to that of AA index, except that the common neighbors were weighted differently ($\frac{1}{k}$).

#### 2.2.2. Methods Based on Paths

These kinds of methods mainly use the path information between two vertexes to calculate their similarities. Zhou et al. considered the contribution of third-order neighbors (path length between vertexes equals 3) based on the common neighbor index (path length between vertexes equals 2) and proposed the Lp (local path) index, which improved the prediction accuracy of CN index [28,29]. Katz et al. considered all possible paths between two vertexes and proposed the famous Katz index [11]. Chen et al. defined the relative strength of each path between two vertexes and proposed RSS index (relation strength similarity), which was applicable to weighted networks [30]. In addition, Papadimitriou et al. proposed the FL (friend link) indicator, which calculated the similarity of vertex pairs by traversing all the bounded path length between two vertexes [31].

#### 2.2.3. Methods Based on Random Walk

This kind of methods uses the transition probability of a vertex to its neighbor vertexes to calculate the probability that the current vertex reaches its destinations with random walk. HT (hitting time) index defined the average number of steps for a particle with a random walk from vertex $v_{x}$ to vertex $v_{y}$ [32]. The less $HT(v_{x},v_{y})$ was, the closer $v_{x}$ and $v_{y}$ were. The SimRank index described the mean time required for two particles starting from $v_{x}$ and $v_{y}$ to encounter [33]. RPR (rooted PageRank) index could be regarded as an expanded application of PageRank [2]. It used the PageRank algorithm to rank the probability of edge formation between vertexes, and considered that the rank of vertex pairs $\left(v_{x},v_{y}\right)$ was proportional to the probability that a particle started from $v_{x}$ and arrived at $v_{y}$. The higher the ranking, the more likely it was to form a link.

### 2.3. Link Prediction Methods Based on Learning

The link prediction methods based on learning mainly use the similarity index, internal attributes of vertexes and external topological information to obtain the feature vectors, and the constraints among the feature vectors are obtained through learning. Researchers mainly study the link prediction methods based on learning from the following four perspectives: feature-based classification methods, probabilistic graph models, matrix decomposition methods, network embedding methods and graph neural networks methods.

#### 2.3.1. Feature-Based Classification Methods

Such methods treat the link prediction problem as a binary classification problem, and use the intrinsic properties of vertexes and the external structure information to obtain feature vectors, then use them to learn and obtain the classification results. Li et al. proposed a kernel-based graph learning method that used the users’ age, education level, book titles, keywords and other characteristics to predict the formation of user-book links [34]. Pujari and Kanawati proposed a new binary topological link prediction method for political and election-related social networks [35]. Based on the equilibrium theory [36] of network structure, Chiang et al. proposed a link prediction method based on supervised learning, which was suitable for labeled networks [37]. Wu et al. proposed an interactive learning framework that transformed linked prediction problems into factor graph model solutions [38]. Lichtenwalter et al. proposed the vertex collocation profile (VCP) method by combining the topological structure methods with the supervised learning methods [17,18].

#### 2.3.2. Probabilistic Graph Models

Such methods suggest that the possibility of forming a link between two vertexes can be expressed by a probability value, such as the topological similarity of random walk or the transfer probability. Clauset et al. proposed a simple hierarchical model HSM (hierarchical structure model) that implemented link prediction by obtaining the optimal network hierarchy [39]. This method was excellently predictive, but the computational overhead was too large. The stochastic block model divided the vertexes in a network into groups, and the probability value of whether two vertexes were linked depended on the group in which the vertexes were located [40]. In other words, the status of vertexes in the same group was the same. Kashima et al. proposed a parametric probabilistic model for network evolution that could be used for link prediction of dynamic networks [41].

#### 2.3.3. Matrix Decomposition Methods

Menon et al. treated the link prediction problem as a matrix completion problem and used an extended matrix decomposition method to solve the link prediction problem [42]. This approach solved the problem of network imbalance and was suitable for networks where there were far more negative links than positive links.

#### 2.3.4. Network Embedding Methods

Perozzi et al. presented DeepWalk [43], a novel approach for learning latent representations of vertexes in a network. These latent representations encoded social relations in a continuous vector space, which was easily exploited by statistical models. Grover et al. proposed node2vec, an algorithmic framework for learning continuous feature representations for vertexes in networks [15]. In node2vec, they learned a mapping of vertexes to a low-dimensional space of features that maximized the likelihood of preserving network neighborhoods of vertexes. They defined a flexible notion of a vertex’s network neighborhood and designed a biased random walk procedure, which efficiently explored diverse neighborhoods.

#### 2.3.5. Graph Neural Networks Methods

Each method mentioned above has a strong assumption on when two vertexes are likely to link, which limits their effectiveness on networks where these assumptions fail. In this regard, a more reasonable way should be learning a suitable heuristic from a given network instead of using predefined ones. Therefore, Zhang et al. studied a heuristic learning paradigm for link prediction [44]. First, they developed a novel γ-decaying heuristic theory. The theory unified a wide range of heuristics in a single framework, and proved that all these heuristics could be well approximated from local subgraphs.

GNN is able to learn hidden features from graphs which can be used for link prediction task in graphs. Link predictions based on GNNs have gained much attention of researchers due to their convincing high performance in many real-world graphs. Islam et al. studied some similarity and GNN-based link prediction approaches in the domain of homogeneous graphs that consisted of a single type of (attributed) vertexes and single type of pairwise links [45], and achieved promising results against several benchmark graphs.

### 2.4. Link Prediction Methods Based on Social Theory

Different from other link prediction methods, the link prediction methods based on social theory consider the interactive information between people, and improve the accuracy and interpretability of link prediction.

Valverde-Rebaza and Lopes, based on fully considering the interests and habits of Twitter users, combined community information with topological structure information to predict the possible future links in Twitter [46]. Experiments showed that this method was effective for link prediction of large, heterogeneous, and directed social networks. Liu et al. proposed a link prediction model based on weak links and three types of vertex centrality (degree centrality, proximity centrality and media centrality) [47]. Li et al. considered the “richer are richer” theory [48], pointing out that vertexes in the network not only tended to link with similar vertexes, but also tended to link with central vertexes, and then proposed a series of link prediction methods based on the maximum entropy random walk. Experiments had shown that the prediction effect of this method was better than that of the general random walk methods. Qiu et al. considered the information cascade theory [49] and proposed a link prediction model based on the evolution of vertex behavior and network event-driven framework, and used the locality and connectivity of vertexes to predict the formation of new links [50]. Kashima et al. considered the theory of homogeneity and proposed a semi-supervised link prediction method based on label propagation technology, arguing that if there was a certain link between two vertexes, then there might also be such a link between vertexes that were similar to these two vertexes [51]. This approach was suitable for multi-relational, time-series networks. Ma et al. applied Nash equilibrium theorem [36] to implicit link prediction for the first time, producing a network model based on common neighbors [52]. Experiments had shown that this method was significantly superior to the ordinary common neighbor methods. In addition to the social theory involved in the above methods, social balance and degree distribution are also often used in the field of link prediction and have proven to be very effective.

### 2.5. The Study of Topological Structure

By analyzing the topology information, researchers can not only obtain the local structure information of vertexes, but also infer the environmental attributes of vertexes in the network as well as the structure information of the network, and then analyze the nature and characteristics of the network.

Chandrasekhar et al. proposed a seed graph generation model SUGMs (subgraph generation models) [53], which believed that networks were composed of a variety of sub-graphs, such as links, triangles, stars, etc. Juszczyszyn et al. believed that the distribution of subgraphs in complex networks was stable and would not change dramatically with the drastic changes in network structure [54,55]. Therefore, the statistical data of subgraph evolution could be used to describe the change in network structure. They proposed the TTM method (triad transition matrix) based on a network subgraph [56], which analyzed the dynamic evolution of the network by using the change of the number of 3-vertex subgraphs, and predicted the formation of links by using the evolution information of 3-vertex graphs. Compared with the common neighbors method and the preference attachment method, the proposed method could maintain a good link prediction effect even when the network changed dramatically.

## 3. Problem Statement

Social networks can be divided into directed networks (e.g., Twitter) and undirected networks (e.g., Facebook) according to whether their edges are directed. They can be divided into static networks and dynamic networks according to whether they have temporality. Static networks refer to the networks whose topology information does not change with time. In this kind of network, our link prediction task refers to the prediction of unseen links, namely, links that exist in the network but have not been found. Dynamic networks $N=\{N_{1},N_{2},\cdots ,N_{T}\}$ refer to the networks which are sets of ordered graphs in time, among which $N_{t}=<V_{t},E_{t}>$ is the network topology at the moment *t*, $V_{t}$ and $E_{t}$ are the vertex set and edge set at the moment, respectively. In this kind of network, our link prediction task is to predict the future links that will appear at moment $t^{{}^{\prime }}\left(t^{{}^{\prime }}\geq t\right)$ according to the information at present.

Consider a social network G(V,E) at moment $t_{0}$, where *V* is the set of vertexes and *E* is the set of edges. The universal set *U* represents all possible $\frac{\left|V|\times (|V|-1\right)}{2}$ links, where |V| denotes the number of elements in set *V*. Then the set of nonexistent links is U−E. The problem is to find all possible links in U−E in the future at moment $t_{1}\left(t_{1}\geq t_{0}\right)$ or links that we lost at this moment $t_{0}$. Note in general, link prediction problems assume that vertexes in the graph exist statically, namely, link prediction does not take into account new or missing vertexes in the graph.

**Figure 1:**
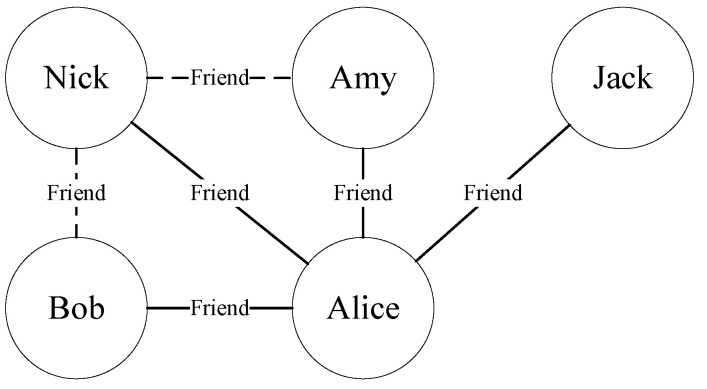
An example of link prediction.

A social network composed of five people can vividly explain this problem. As shown in Figure 1, the solid lines represent the existing relationship at time $t_{0}$, and the dashed lines represent the new relationship formed between the intervals $\left[t_{0},t_{1}\right]$. At time $t_{0}$, Alice and Bob are friends, and Alice is Nick’s friend, too. Maybe Alice will introduce Bob to Nick, so at time $t_{1}$, Bob and Nick will become friends. Similarly, Nick and Amy may become friends at time $t_{1}$.

**Figure 2:**
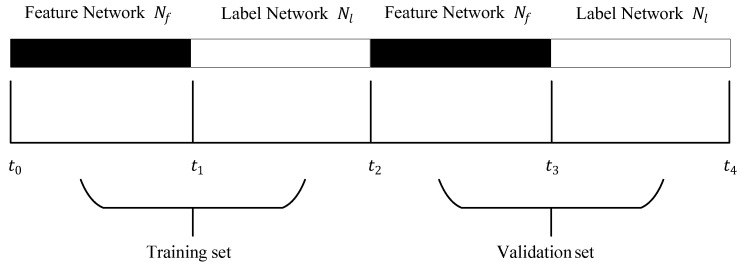
The link prediction dataset is divided into training set and validation set, and the network *N* in each set is divided into Feature Network $N_{f}$ and Label Network $N_{l}$.

As shown in Figure 2, the dataset $\left(t_{0},t_{4}\right)$ is divided into training set $\left(t_{0},t_{2}\right)$ and validation set $\left(t_{2},t_{4}\right)$. The training set is used to generate the model, and the validation set is used to verify the effectiveness of the algorithm. Since the prediction of future links will appear in the next stage, we divide the network *N* both in the training set and the validation set into two parts, called Feature Network $N_{f}$ and Label Network $N_{l}$. For example, in the training set, the generated network in the current time $\left(t_{0},t_{1}\right)$ is called Feature Network $N_{f}$ and the network generated in the future time $\left(t_{1},t_{2}\right)$ is called Label Network $N_{l}$ $\left(t_{2}\geq t_{1}\geq t_{0}\right)$. Feature Network $N_{f}$ is used to generate feature vectors of vertex pairs, and then we can classify feature vectors according to whether the pairs of vertexes in Label Network $N_{l}$ will form links or not. It is also similar for the validation set.

According to the connection of vertexes in the network, we can divide the vertex pairs into different sets.

**Definition** **1** (Unlinked edge set). *For a Feature Network $N_{f}$, this paper denotes edges that belong to U but do not belong to $E_{N_{f}}$ by unlinked edge set $E_{ul}$.*

(1) $$E_{ul}=\left\{\left(x,y\right)|\left(x,y\right)\in U\wedge \left(x,y\right)\notin E_{N_{f}}\right\}.$$

**Definition** **2** (Nonexistent edge set). *For a Feature Network $N_{f}$, this paper denotes edges that belong to unlinked edge set $E_{ul}$ but do not belong to the edge set $E_{N_{l}}$ by nonexistent edges $E_{ue}$.*

(2) $$E_{ue}=\left\{\left(x,y\right)|\left(x,y\right)\in E_{ul}\wedge \left(x,y\right)\notin E_{N_{l}}\right\}.$$

The link prediction needs a score $S_{xy}$ for each pair of vertexes $\left(x,y\right)\in E_{ul}$. Then, all unconnected vertex pairs are sorted in descending order according to the score value, so that the first vertex pair in the list has the highest probability of forming edges.

**Figure 3:**
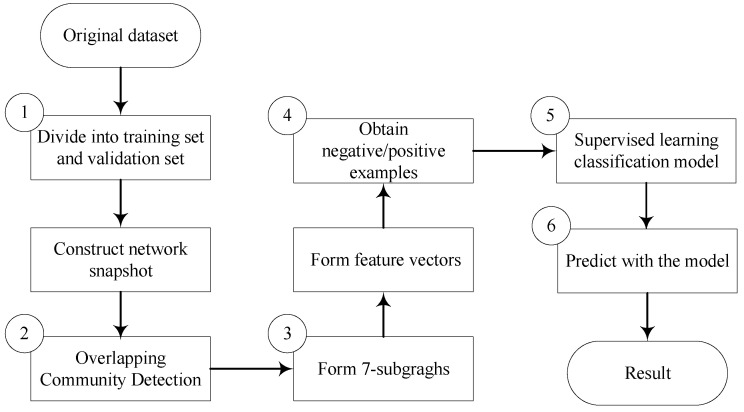
The framework of the supervised link prediction method using OVCP.

In this paper, community detection algorithms were used to select the vertex pairs that need to be predicted, and the OVCP method was used to record the topological structure information of the paths with length less than or equal to six between vertex pairs. Combined with the supervised learning method, a new link prediction method based on topological structure was proposed. As shown in Figure 3, the framework of our method is mainly divided into the following six steps:

(1) The training set and validation set are selected from the original dataset, and the network snapshot is constructed according to the edge information of the training set and validation set;
(2) The overlapping community detection algorithms are applied to divide the network into multiple communities;
(3) For unconnected vertex pairs in each community, we find all paths with length less than or equal to 6 between two vertexes and record the transition vertexes on the paths. Then we complete the paths between vertexes to form 7-subgraghs. For each 7-subgraph, its address is calculated, and the number of occurrences of each 7-subgraph is counted to form the feature vector;
(4) For each vertex pair obetained in step (3), if it appears in the Label Network $N_{l}$ then denoting as 1, otherwise denoting as −1. Then a positive sample or a negative sample can be obtained by combining the feature vector of this vertex pair obtained in step (3) and its label;
(5) The positive and negative samples sets obtained in step (4) will be fed to the supervised learning classification model as training data;
(6) Validate performance on validation set with the trained model obtained in step (5).

Finally, we give the symbols and fundamental definitions used throughout the paper as Table 1 lists.

## 4. OVCP Feature Extraction

In this section, we first introduce the basic concepts of VCP and its drawbacks. To address these issues, this paper proposes a new link prediction method, OVCP, and details its basic principles.

### 4.1. VCP

Lichtenwalter et al. proposed the vertex collocation profile (VCP) method by combining the topological structure methods with the supervised learning methods [17,18]. This method makes full use of the rich topology information between vertexes. By statistical analysis of the types and numbers of the subgraphs composed of the vertex pair $\left(v_{x},v_{y}\right)$ and other arbitrary n−2 vertexes, where *n* is the number of vertexes in the subgraph, the relation vector of this vertex pair can be obtained. In this way, after the relation vectors of all vertex pairs are obtained, they are taken as the feature vectors of machine learning. The supervised learning method is used to train the supervised learning model and predict the formation of links.

Specifically, the VCP method thinks any n−2 vertexes are likely to form n-subgraph with vertex pair $v_{x}$ and $v_{y}$. To represent all possible subgraphs of an n-subgraph, the VCP method records each subgraph using an adjacency matrix.

**Definition** **3** (N-subgraph). *The network consists of vertex pair $v_{x}$ and $v_{y}$ and other n−2 vertexes is called n-subgraph.*

**Figure 4:**
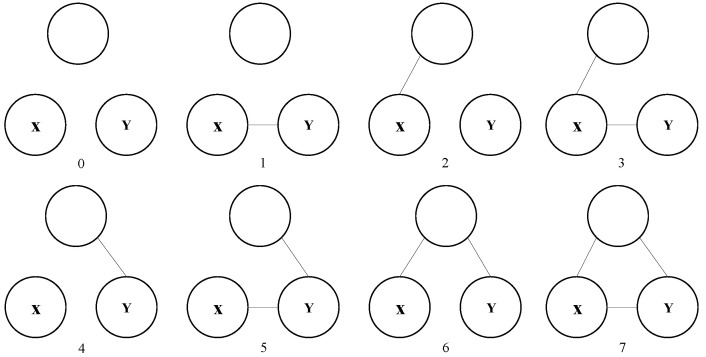
All possible subgraphs when the number of vertexes is 3.

Figure 4 shows all possible subgraphs in *n*-subgraph when n=3. There are 8 subgraphs.

**Definition** **4** (VCP Isomorphism). *For subgraph G(V,E) and $G^{{}^{\prime }}\left(V^{{}^{\prime }},E^{{}^{\prime }}\right)$, if G and $G^{{}^{\prime }}$ are VCP-isomorphic, there exists a bijective mapping f from V to $V^{{}^{\prime }}$, where f has the following property: for any a,b∈V, (a,b)∈E if and only if $\left(f\left(a\right),f\left(b\right)\right)\in E^{{}^{\prime }}$.*

**Figure 5:**
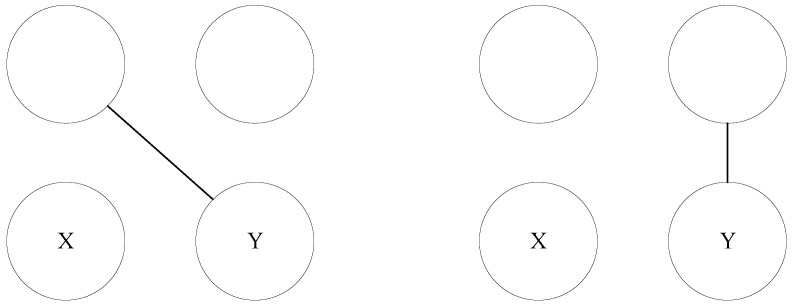
An example of VCP isomorphism.

Note that VCP isomorphism exists between some subgraphs of N-subgraphs. For example, in Figure 5, these two subgraphs are VCP isomorphic, so it is entirely reasonable to assume these two subgraphs are the same graph.

### 4.2. OVCP

The VCP method believes that any vertex will form a subgraph with another vertex and influence the formation of the corresponding link, but there is no practical reason for this setting. In a social network, every user is a unique individual, whose choice is influenced by his friends and family, and in turn affects the choice of his friends and family.

According to the “three degrees of influence rule” [19], if the distance between users exceeds three degrees, the influence will gradually disappear. Therefore, the prediction of links between users only needs to consider the influence of vertexes within three degrees from the user. However, this is only the results of statistics and observation. There is no guarantee that vertexes beyond the three degree will not affect the link prediction, which will affect the performance. The OVCP (optimized vertex collocation profile) method improves the VCP method by avoiding the vertexes that have no influence on the vertex to be predicted, namely, the vertexes that are more than three degrees away from the vertex to be predicted. Moreover, the OVCP method also avoids those vertexes who have no connection to one vertex of the vertex pair to be predicted, namely, vertexes that cannot arrive at the pair to be predicted at the same time. Because these vertexes will not influence the information communication between vertexes to be predicted, and will not have a positive impact on the prediction of the links between them.

Specifically, the OVCP method avoids those vertexes that have no influence on the vertex pair to be predicted and those vertexes that are only related to one vertex of the pair to be predicted by obtaining transition vertexes.

Inspired by “Six Degrees of Separation” [20], we consider the subgraph whose path length is no longer than six between vertex pairs, which means there are at most seven vertexes on the path and we call this subgraph the *7-subgraph*.

**Definition** **5** (Transition vertex). *If a link may exist between vertex $v_{x}$ and $v_{y}$, meanwhile, the distance $l(k,v_{x})$ between vertex k and $v_{x}$ and the distance $l(k,v_{y})$ between vertex k and $v_{y}$ subject to inequality constraint $l\left(k,v_{x}\right)+l\left(k,v_{y}\right)\leq 6$, we call the vertex k transition vertex in the path between vertex $v_{x}$ and $v_{y}$.*

**Definition** **6** (7-subgraph). *If the number of transition vertexes on the path between vertex $v_{x}$ and vertex $v_{y}$ is 5, we call the subgraph consists of $v_{x},v_{y}$ and these transition vertexes and edges between them 7-subgraph.*

Here ’7’ refers to the number of vertexes in the subgraph. However, in the actual experiments, the number of transition vertexes in the vertex pair to be predicted is usually less than 5. For the convenience of calculating the address of 7-subgraph, we artificially add the corresponding number of free vertexes to the subgraph with less than 7 vertexes. These free vertexes do not have any effect on the calculation of the 7-subgraph address.

7-subgraphs are stored in the form of adjacency matrices. The adjacency matrix of 7-subgrapgh G(V,E) is defined as a 7×7 matrix **D** with elements *d*, where the definition of $d_{ij}$ is as follows:

(3) $$d_{ij}=\left\{\begin{array}{c} 1,\;(v_{i},v_{j})\in E\\ 0,\;(v_{i},v_{j})\notin E, \end{array}\right.$$

where 1≤i,j≤7. In order to obtain a unique address for each 7-subgraph, a *bit matrix* of 7-subgraph is defined to distinguish edges in the 7-subgraph so that each edge is given a different weight. Then take the inner product of the adjacency matrix of the 7-subgraph with the bit matrix to obtain the address of the 7-subgraph. In this way, we can not only distinguish different 7-subgraphs, but also derive the formation of edges in the 7-subgraph according to the address of the 7-subgraph.

**Definition** **7** (Bit matrix of 7-subgraph). *The bit matrix **V** of an undirected graph G with seven vertexes is a matrix of order 7×7. Let the set of all elements of the matrix be N, then **V** is defined as follows:*

(4) $$V=v_{ij}\in N|v_{ij}=\left\{\begin{array}{c} 2^{\left(7\left(i-1\right)-\frac{1}{2}\left(i-1\right)\left(i+1\right)+j-2\right)},if\;i\leq j\\ 0\;\;\;\;\;\;\;\;\;,if\;i=j\\ 2^{\left(7\left(j-1\right)-\frac{1}{2}\left(j-1\right)\left(j+1\right)+i-2\right)},if\;i\geq j \end{array}\right.$$

In other words,

(5) $$V=\left[\begin{array}{ccccccc} 0 & 1 & 2 & 4 & 8 & 16 & 32\\ 1 & 0 & 64 & 128 & 256 & 512 & 1024\\ 2 & 64 & 0 & 2^{11} & 2^{12} & 2^{13} & 2^{14}\\ 4 & 128 & 2^{11} & 0 & 2^{15} & 2^{16} & 2^{17}\\ 8 & 256 & 2^{12} & 2^{15} & 0 & 2^{18} & 2^{19}\\ 16 & 512 & 2^{13} & 2^{16} & 2^{18} & 0 & 2^{20}\\ 32 & 1024 & 2^{14} & 2^{17} & 2^{19} & 2^{20} & 0 \end{array}\right].$$

**Definition** **8** (Address of 7-subgraph). *The address of subgraph can be obtained from the inner product of adjacency matrix and bit matrix:*

(6) $$\psi \left(G\right)=\sum_{i=1}^{7}\sum_{j=1}^{7}\left(v_{ij}\times d_{ij}\right).$$

Since the adjacency matrix **D** of an undirected graph is symmetric about the diagonal, the bit matrix is also symmetric about the diagonal. Then the address ψ(G) is equal to twice the address $\psi (G^{{}^{\prime }})$, where $G^{{}^{\prime }}$ is the part above the diagonal. If the bijection from $D^{{}^{\prime }}$ to address $\psi (G^{{}^{\prime }})$ exists, there exists bijection from adjacency matrix **D** to address $\psi (G^{{}^{\prime }})$ since there is a one-to-one correspondence between $D^{{}^{\prime }}$ and **D**. Thus, there exists bijection from **D** to address ψ(G).

In fact, because the part above the diagonal of the adjacency matrix $D^{{}^{\prime }}$ marks whether each bit is 0 or 1, the process of generating the address of adjacency matrix can be transformed to generate a 21-bit binary number. Each element of the bit matrix from left to right and top to bottom corresponds to a decimal value. Make inner product of the part above the diagonal of adjacency matrix and the part above the diagonal of bit matrix, then we can obtain a binary number corresponding to the part above the diagonal of adjacency matrix. The binary numbers would be different as long as the part above the diagonal of adjacency matrix is different. Therefore, there exists bijection from $D^{{}^{\prime }}$ to $\psi (G^{{}^{\prime }})$ and bijection from **D** to ψ(G). So there is a one-to-one correspondence between adjacency matrix **D** and ψ(G).

According to the definition of VCP Isomorphism, the subgraphs obtained by switching transition vertexes are VCP isomorphism, but these subgraphs have different subgraph addresses. If two VCP isomorphic subgraphs both appear once, then we cannot represent the two subgraphs through the relation vectors as the same subgraph appearing twice. So we need to make the two subgraphs have the same address. We use the concept of *minimal address*. All VCP-isomorphic subgraphs have the same minimal address. Representing these subgraphs with the minimal address allows these subgraphs to be represented as the same through the relation vectors.

**Definition** **9** (Minimal Address). *Swap the transition vertexes in the subgraph arbitrarily, and calculate the address of the generated subgraph. The smallest of these addresses is the minimum address of the subgraph.*

(7) $$\psi_{min}\left(G\right)=argmin\left(\sum_{i=1}^{7}\sum_{j=1}^{7}\left(v_{ij}\times d_{k_{3,7}^{p}\left(i\right),k_{3,7}^{p}\left(j\right)}\right)\right)$$

*where $k_{3,7}^{p}\left(i\right)$ denotes union of the $p_{th}$ permutation of set {3,4,5,6,7} and set {1,2}.*

There are 120 permutations of the five transition vertexes, and the minimal address of the 7-subgraph is the smallest one of the 120 subgraphs.

With the definition of minimal address, we can obtain the relation vector of each vertex pair. Firstly, record vertex pairs whose path length less than or equal to 6 and preserve the transition vertex for each vertex pair. Then complete the links between vertexes on each path between vertex pairs and the completed topology structure is recorded with the 7-subgraph. For vertex pair $\left(v_{x},v_{y}\right)$ which is possibly linked in the future, we can obtain a relation vector. One dimension of the vector is the minimal address of one kind of 7-subgraph, its value is the number of occurrences of such kind of 7-subgraph in the path between vertex $v_{x}$ and $v_{y}$. This vector is used to keep the dependency among transition vertexes as well as between transition vertexes and vertex $v_{x}$ or $v_{y}$.

**Definition** **10** (Relation vector). *Assume n is the number of vertexes in n-graph and $|G^{n}|$ is the number of all possible subgraphs. Relation vector $VCP_{x,y}^{n}$ is a $|G^{n}|$-dimensional vector. The element $VCP_{x,y}^{n}\left(i\right)$ in the relation vector is equal to |ψ(i)|, where $i=0,1,2,\cdots ,|G^{n}|-1$, |ψ(i)| represents the number of occurrences of subgraphs whose address is ψ(i).*

**Figure 6:**
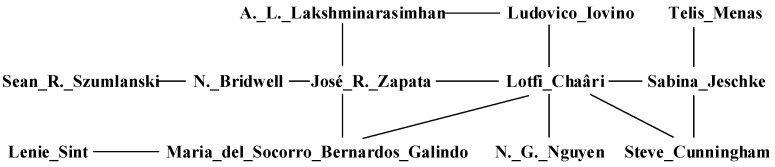
A subgraph from DBLP dataset.

For instance, Figure 6 is a part derived from real academic collaboration network. Each vertex represents a researcher, and each edge indicates that there is academic cooperation between the two researchers.

**Figure 7:**
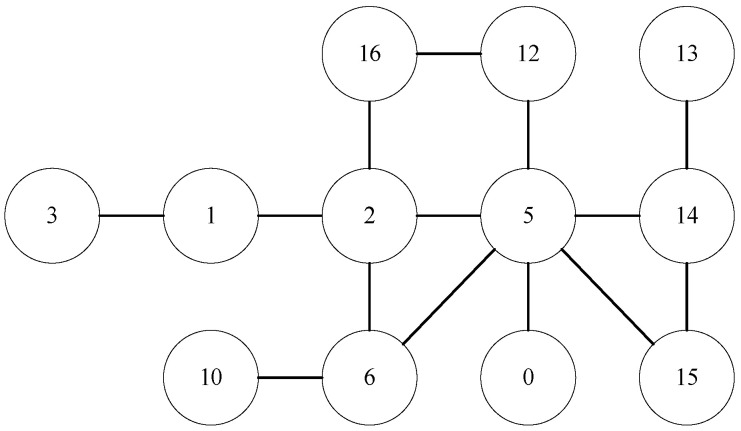
Abstract vertexes corresponding Figure 5.

**Figure 8:**
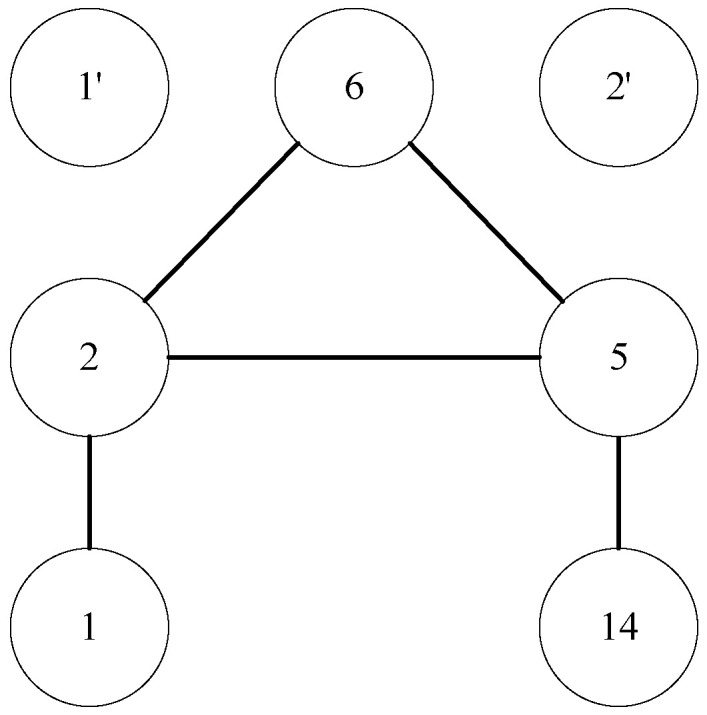
7-subgraph corresponding to path 1-2-6-5-14.

For convenience, we transform Figure 6 to a graph with abstract vertexes. As shown in Figure 7, if the vertexes to be predicted are vertex 1 and vertex 14, 1-2-6-5-14 is one path between them. The corresponding 7-subgraph is showed in Figure 8.

We simply set vertexes to be predicted as $v_{1}$ and $v_{2}$, and transition vertexes as $v_{3}∼v_{7}$. Then vertex 1 corresponds to $v_{1}$, vertex 14 corresponds to $v_{2}$, vertex 2 corresponds to $v_{3}$, vertex 5 corresponds to $v_{4}$, vertex 6 corresponds to $v_{5}$, free vertex $1^{{}^{\prime }}$ corresponds to $v_{6}$, and free vertex $2^{{}^{\prime }}$ corresponds to $v_{7}$. Thus the 7-subgraph can be represented by the following adjacency matrix:

(8) $$\left[\begin{array}{ccccccc} 0 & 0 & 1 & 0 & 0 & 0 & 0\\ 0 & 0 & 0 & 1 & 0 & 0 & 0\\ 1 & 0 & 0 & 1 & 1 & 0 & 0\\ 0 & 1 & 1 & 0 & 1 & 0 & 0\\ 0 & 0 & 1 & 1 & 0 & 0 & 0\\ 0 & 0 & 0 & 0 & 0 & 0 & 0\\ 0 & 0 & 0 & 0 & 0 & 0 & 0 \end{array}\right].\;$$

**Figure 9:**
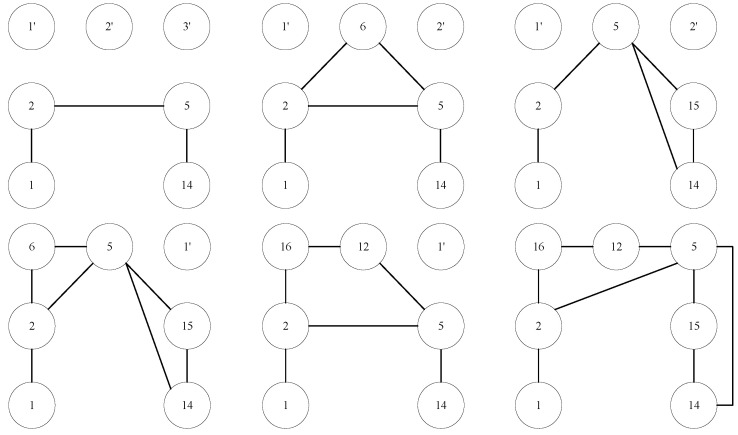
Subgraphs corresponding to possible paths between vertex 1 and vertex 14.

Then the address of 7-subgraph in Figure 8 is 2+128+2048+4096+32768+2+128+2048+4096+32768=78084. In Figure 6, there exists 6 paths between vertex 1 and vertex 14. The corresponding subgraph for each path is shown in Figure 9.

(1) 1-2-5-14
(2) 1-2-6-5-14
(3) 1-2-5-15-14
(4) 1-2-6-5-15-14
(5) 1-2-16-12-5-14
(6) 1-2-16-12-5-15-14

According to definition of minimal address of 7-subgraphs, the minimal address of the six subgraphs are respectively 4232, 77,960, 12,688, 95,368, 221,328, 256,144. Then we can obtain the relation vector of vertex 1 and vertex 14 since each address just occurs once then the value of each feature is 1. $v_{1}v_{2}$ = (4232:1, 77,960:1, 12,688:1, 95,368:1, 221,328:1, 256,144:1), where (*x*:1) means the element corresponding to address *x* in the relation vector is 1.

In general, the OVCP method extends the number of vertexes in one subgraph to 7, which can take use of more topological structure information. In the selection of subgraphs, We believe that the information is transmitted along the path, which avoids those subgraphs where there is no path between the vertexes to be predicted, and also reduce the complexity.

### 4.3. Complexity Analysis

If we use VCP method to predict links, the time complexity of feature extracting is $\Omega ({\left(\frac{\left|E\right|}{\left|V\right|}\right)}^{n-2})$. The time complexity of OVCP method mainly comes from the computing all possible path length less than or equal to 6. The average number of neighbors of every vertex is $\frac{\left|E\right|}{\left|V\right|}$. Finding a path of length 6 requires traversing 5 levels, thus average time complexity is $\Omega ({\left(\frac{\left|E\right|}{\left|V\right|}\right)}^{5})$. In the worst case (i.e. the network N is a fully connected network of order n), the time complexity is $O(|V{|}^{5})$. Therefore, the OVCP method has lower time complexity.

## 5. Community Detection

This section introduces the basic principles of the two community detection algorithms, compares the differences between them, and explains how to use these two algorithms to divide the network into multiple communities to reduce the complexity of link prediction.

### 5.1. Fast Unfolding Community Detection

Modularity is a measure of the strength of the structure of the network community [32,33], and its formal definition is given below:

**Definition** **11** (Modularity). *Given network N and its division of vertex sets, Modularity Q can be difined as:*

(9) $$Q=\frac{1}{2m}\sum_{i,j}\left[A_{i,j}-\frac{k_{i}k_{j}}{2m}\right]\delta \left(c_{i},c_{j}\right)$$

(10) $$k_{i}=\sum_{j}A_{i,j}$$

(11) $$m=\frac{1}{2}\sum_{i,j}A_{i,j},$$

*where $A_{i,j}$ denotes the weight of edge between vertex i and vertex j; $k_{i}$ denotes the sum of the weights of all edges connecting vertex i; m denotes the sum of weights of all edges; $c_{i}$ denotes the subnet number to which vertex i belongs; δ(u,v)=1 if and only if u=v.*

The fast unfolding community detection algorithm optimizes the objective function until convergence by adjusting the community number to which each vertex belongs, and outputs the community detection results.

The process of the algorithm is as follows: we first traverse each vertex and temporarily modify the community number of the vertex to its adjacent vertexes, then calculate the modularity increment and use the modification of the non-negative increment as the final modification. We continuously perform the above steps until the modularity converges. After convergence, the vertexes with the same community number are merged into the same vertex. In the network composed of new vertexes, the edge weights are calculated by the sum of the edge weights between the communities [32,33].

However, the communities generated by this community detection algorithm do not overlap, and only consider the links within the community, ignoring the links between communities.

### 5.2. Overlapping Community Detection

To address the above problem, this paper uses an edge-community-based overlapping community detection algorithm which proposes the concept of edge-community, which regards the community as a collection of closely connected edges [31].

The algorithm consists of two phases: (1) we first traverse each vertex and combine all the edges connected to this vertex in pairs. Then we compare the similarity of each pair of edges, and add the similarity and the three vertexes of these two edges into list *L* as a quadruple. After all quadruples are added, sort the list *L* by the similarity; (2) We traverse the quadruple in list *L*. For each quadruple, judge whether the current similarity *s* is less than the threshold or not. If it does, stop the mergence and directly assign the current partition *p* to bestP. Otherwise, merge the communities where the two edges are and perform the calculation of the relevant variable values. After the traversal is completed, bestP is the result set detected by the edge community. This method needs to calculate the similarity of any two edges in the edge set, and then merge the communities by traversing all edge pairs. So the complexity of the algorithm is $O(|E{|}^{2})$.

**Figure 10:**
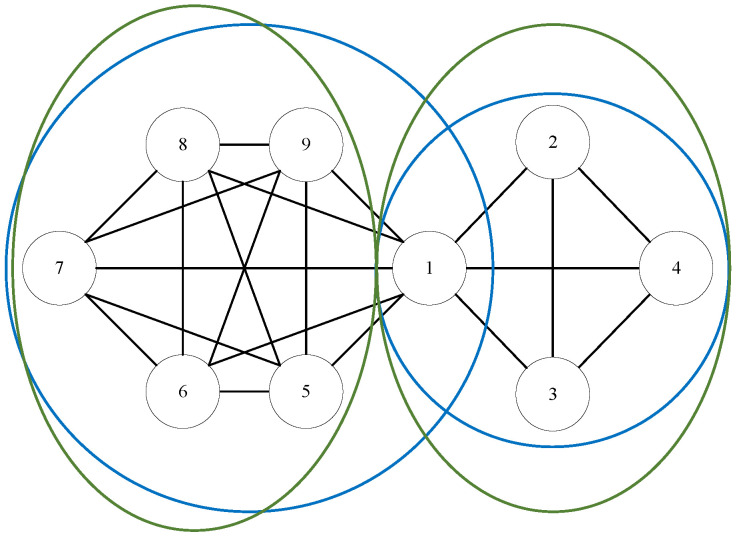
Difference between overlapping community detection algorithm and fast community detection algorithm.

Using the overlapping community detection algorithm, the network can be divided into several small overlapping communities, and we limit the link prediction to within the community, which can reduce the complexity. This algorithm takes into account links between non-overlapping communities by transferring the links between non-overlapping communities to the links within overlapping communities. As shown in Figure 10, (1, 2, 3, 4) and (5, 6, 7, 8, 9) are the results of non-overlapping community detection algorithms circled by green circles while (1, 2, 3, 4) and (1, 5, 6, 7, 8, 9) are the division results of overlapping community detection algorithm circled by blue circles. The prediction using the non-overlapping community detection algorithm will ignore the link formed between vertex 1 and vertex 8, while the overlapping community detection algorithm will predict whether vertex 1 and vertex 8 will form a link.

## 6. Learning-Based Link Prediction Model

The process of training the model is as follows: apply the OVCP method to each vertex pair in the unlinked edge set $E_{ul}$ of the Feature Network $N_{f}$ in the training set to generate feature vectors. Then count the link formation of each vertex pair in the Label Network $N_{l}$ in the training set. The vertex pair that forms the link is taken as a positive sample, denoted as class 1, and the vertex pair that does not form a link is taken as a negative sample, denoted as class −1. Therefore, a category sequence is generated. Then use the feature vector sequence *X* and the category sequence *Y* as training data to train the classification model.

The process of evaluating the validation set is as follows: apply the OVCP method to each vertex pair in the unlinked edge set $E_{ul}$ of the Feature Network $N_{f}$ in the validation set to generate feature vectors. Then feed these feature vectors to the model obtained by supervised learning training, and obtain the score sequence $Y_{Score}$ of feature vectors. For example, the support vector machine (SVM) model uses the distance sequence of the feature vectors from the hyperplane as $Y_{Score}$, and the naive Bayes (NB) classifier model uses the vectors as the probability sequence of the positive class as $Y_{Score}$. Then the statistical validation is concentrated on whether each vertex pair in the Label Network $N_{l}$ forms a link or not. The vertex pair that forms the link is taken as a positive sample, marked as class 1, and the vertex pair that does not form a link is regarded as a negative sample, marked as class −1. The AUC value can be obtained through the category sequence Y and the score sequence $Y_{Score}$.

## 7. Experiments

In this section, based on the discussion in the previous sections, this paper uses python to implement our link prediction method based on topological structure with some open source toolkits such as LibSVM [57], sklearn [58], numpy [59], scipy [60] and networkx [61]. There are five modules in our method: preprocessing, vertex pair selection, feature extraction, supervised learning and result validation.

We first preprocess the dataset, then select vertex pairs from the training set to build a vertex pair set. For each vertex pair, we generate a feature vector and a set of positive and negative samples. After learning from this set with the supervised learning training model, we can generate feature vectors from the validation set and use this model to obtain the experimental results.

In order to evaluate the classifier’s ability to classify both positive and negative samples and whether the classifier can still make a reasonable evaluation in case of unbalanced samples, we use AUC [62] as the evaluation criteria. The ROC curve is a curve with false negative (FP) as the abscissa and true negative (TP) as the ordinate, and the AUC value is the area under this curve. It can be understood as the probability that the score value of a random selection of an edge from the edge set $E_{N_{l}}$ of the Label Network of the validation set is higher than the score value of random selection of an edge from the nonexistent edge set $E_{ue}$. Therefore, when the AUC value is greater than 0.5, it means that the algorithm is more precise than the random selection method.

### 7.1. Datasets

We chose a bunch of datasets which are easy to structure into an undirected graph. In these experiments, we used real social network datasets, DBLP, Facebook Friendship and Facebook wall.

DBLP is the on-line reference for bibliographic information on major computer science publications. The author’s papers are listed by publication year. The author’s cooperation network can be obtained based on these data, and according to the cooperation network we can predict the author’s future cooperation relationship. We used two sets of data. The first set is the data from 1976 to 1979. The data in 1976 is used as the Feature Network in the training set, the data in 1977 is used as the Label Network in the training set. The data in 1978 is used as the Feature Network in validation set, the data in 1979 is used as the Label Network in the validation set. The second set is the data from 1981 to 1984 and is used in a similar way to the first set of data.

Facebook Friendship is the data on friend relationships in Facebook. This dataset lists the time when friends formed a relationship. Based on the current friend relationship, we can predict the friend relationship that will be formed within a period of time. Facebook Wall is the data of mutual messages between friends in Facebook. This dataset lists the time of messages between friends. We treated the relationships as undirected. According to the current message data, we can predict which pairs of friends will leave messages to each other within a period of time. These two datasets are used similarly to DBLP. The detailed information on the vertexes and edges of datasets is shown in Table 2.

### 7.2. Results

In these experiments, the influence of the parameter selection of the overlapping community detection algorithm and the vertex pair selection methods on the prediction effect of the OVCP method are explored on three datasets, and the OVCP method is compared with other link prediction methods.

#### 7.2.1. Parameter Selection of Overlapping Community Detection

The community detection result of the overlapping community detection algorithm is mainly controlled by one parameter, the similarity threshold. While the current edge similarity is less than the threshold, the mergence is stopped, and the current community division is the community detection result. Therefore, we take the division when the similarity threshold in the range of [0,1] as the community detection result and then apply the OVCP method. The experiment shows the prediction results of different values of the similarity threshold on each dataset.

**Figure 11:**
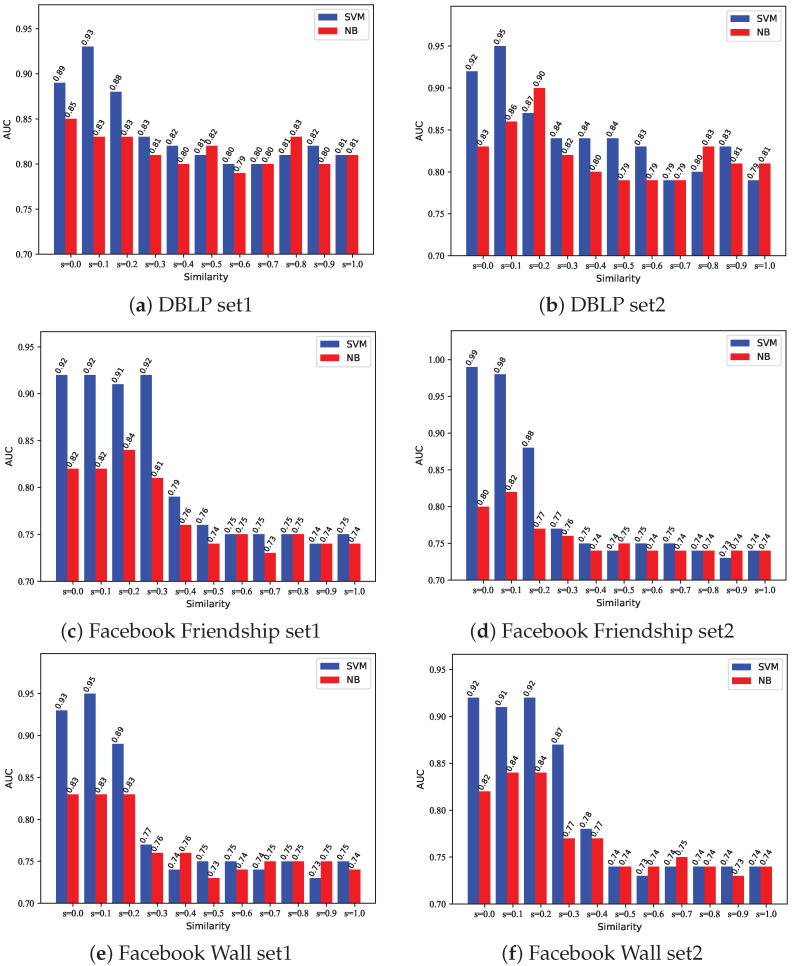
Comparison of prediction results of overlapping community detection algorithms with different parameter selection values.

Figure 11a,b are the prediction results on the DBLP dataset. Figure 11c,d are the prediction results on the Facebook Friendship dataset. Figure 11e,f are the prediction results on the Facebook Wall dataset.

Through observation, we can find that when the SVM and NB are used, the trend of AUC is basically the same. Overall, the SVM method is better than the NB method, which is probably because there are more linear and inseparable characteristics in datasets. As the threshold increases, the size of the community decreases, and the AUC of both methods increases first and then decreases. The reason for the improvement in model performance is that proper merging of edges is beneficial for model learning. The reason for the decline in model performance is that the community is too small at this time, resulting in all links are generated between communities, but our method only considers the links within the community, so the AUC value is very small at this time. Therefore, this paper adopts the method of controlling the similarity threshold to control the partition size, and achieves the best prediction effect by taking the similarity threshold *s* around 0 to 0.2.

#### 7.2.2. Effect of Selecting Vertex Pairs

The selection of vertex pairs methods mainly includes: (1) The vertexes of the entire network are combined in pairs, hereinafter referred to as the Base method; (2) The fast unfolding community detection algorithm is used to divide the network into multiple communities, and then the vertexes in each community are combined in pairs, hereinafter referred to as the CD method; (3) The overlapping community detection algorithm is used to divide the network into multiple communities, and then the vertexes in each overlapping community are combined in pairs, hereinafter referred to as the OCD method. We used three different vertex selection methods combined with the OVCP method to conduct experiments on three different datasets, and compared the prediction results.

**Figure 12:**
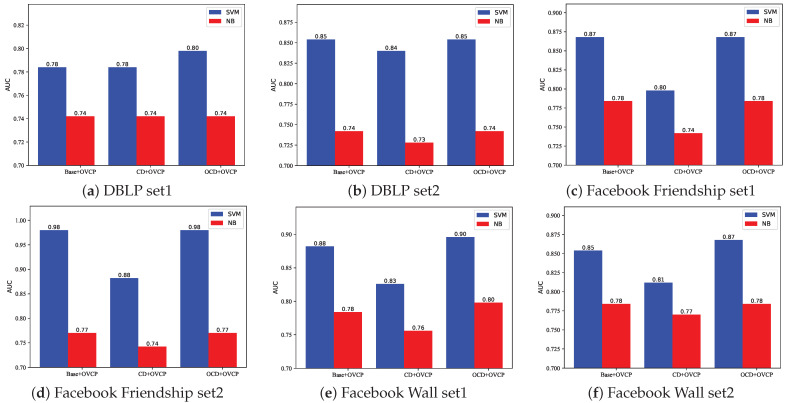
Comparison of prediction effect of three different methods of selecting vertex pairs combined with OVCP method.

From Figure 12a, we can see that, on the DBLP set1, using the Base method and the CD method to select vertex pairs has similar results. The OCD method is slightly better than the other two methods. On the premise of using the SVM method, the prediction effect is significantly better than the NB method.

From the other figures, we can see that the prediction effect of the Base method is better than that of the CD method, but slightly worse than the OCD method, and the prediction effect of the SVM method is also obviously better than NB method.

In general, the Base method is better than the CD method, and the OCD method is better than the other two methods. The reason why the Base method is better than the CD method is that the CD method divides the network into multiple non-overlapping communities and only considers the links formed within the communities, while ignoring the links between the communities. Although the cohesion of the community is guaranteed, the links between communities have been lost. The OCD method not only considers the cohesion of the community, but also considers the overlap between the communities. The number of vertexes removed by overlapping communities detection algorithm is small, which happens to remove those vertex pairs that may be misjudged by the OVCP method. Therefore, the OCD method performs better than the Base method.

In summary, we finally adopt the OCD method as the vertex pair selection method in this paper.

#### 7.2.3. Comparison Experiments

Since the methods in this paper are derived from the VCP method, this experiment compares our method with the VCP method and common basic indicators, including Katz, AA, RA, CN and Jaccard indicators. Our method adopts the OCD vertex pair selection method, selects the corresponding community division, combined with the OVCP method, and uses SVM or NB for supervised learning. The VCP method uses LPMade provided by Lichtenwalter et al. in the two cases of n=3 and n=4 [18].

Specifically, OCD+OVCP+SVM and OCD+OVCP+NB are the methods proposed in this paper, which use overlapping community detection algorithm to select vertex pairs, OVCP method to extract features, and SVM or NB for supervised learning link prediction methods.

**Figure 13:**
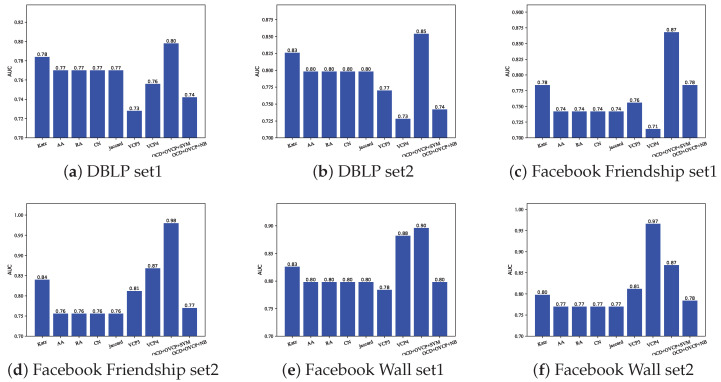
Comparison of prediction effect of three different methods of selecting vertex pairs combined with OVCP method.

As can be seen from Figure 13a, on the DBLP set1, the prediction result of OCD+OVCP+SVM is better than that of VCP method and other basic indicators, while the prediction result of OCD+OVCP+NB method is poor, whose result is worse than most methods. It can be seen from Figure 13b that on the DBLP set2, the prediction results are similar to the former. From the above two prediction results, it can be seen that the performance of the VCP method is even inferior to some basic similar indicators, which may be caused by the network being too small. Among the basic similarity indicators, the Katz indicator uses the path information between the vertex pairs as the scores, which performs better than other basic indicators. However, the Katz method only considers the number of paths and does not consider the complete topological structure information on the path. Therefore, its performance is worse than that of the method proposed in this paper. The AA index is similar to the RA index. The AA index assigns a weight to each vertex according to the degree of common neighbor ($\frac{1}{log(k)}$), where *k* is vertex degree. RA has similar effect with AA index, except that the common neighbors were weighted differently ($\frac{1}{k}$). The Jaccard index is an extension of the CN index. The former considers the ratio of the common neighbor of two vertexes to the union of the neighbors of two vertexes, and the latter only considers the number of common neighbors. In the DBLP datasets, the performance of these four indicators is comparable.

As shown in Figure 13c, on the Facebook Friendship set1, the OCD+OVCP+SVM method and OCD+OVCP+NB perform better than almost all other methods. Among the basic indicators, the Katz indicator performs the best, while other basic indicators are comparable. The performance of VCP method is still not as good as other methods. From Figure 13d, it can be seen that on the Facebook Friendship set2, the OCD+OVCP+SVM method is the best, the OCD+OVCP+NB method performs inferior which is only better than AA, RA, CN and Jaccard. The VCP method performs well. The Katz method performs best in the basic indicators while other basic indicators are comparable.

It can be seen from Figure 13e that the performance of each method on the Facebook Wall set1 is almost the same as that on the Facebook Friendship set2. As can be seen from Figure 13f, on the Facebook Wall set2, the VCP method obtained the best prediction results; the performance of the OCD+OVCP+SVM method is still competitive, which is better than the basic indicators.

In summary, the method proposed in this paper performs well on the three datasets, and is significantly better than the VCP method (n=3 and n=4) on most of the datasets. In addition, the use of SVM for supervised learning has achieved better performance than the use of NB because there are too many inseparable linear characteristics in the data.

#### 7.2.4. Efficiency Experiment

We verify the efficiency on the DBLP dataset, and report the running time of all modules. The specific data are shown in Table 3. It can be seen from the table that the link prediction for all vertex pairs in the network takes a lot of time. With only more than 10,000 vertexes, it takes more than 10,000 seconds to calculate, which is not on the same order of magnitude as other methods.

In theory, the calculation speed of the VCP3 and VCP4 methods is faster, and the complexity is $O\left(\left(\frac{\left|E\right|}{\left|V\right|}\right)\right)$ and $O\left({\left(\frac{\left|E\right|}{\left|V\right|}\right)}^{2}\right)$, respectively. However, the complexity of VCP (n = 7) is $O\left({\left|V\right|}^{6}\right)$, which is the most complicated with the Base+OVCP method.

## 8. Conclusions

This paper implements a link prediction method based on topological structure, which takes social relationships and their formation in Feature Network as the input, and ordering of nonexistent edges as the output. Then, we calculate AUC according to the ordering and the actual formation situation of links in the Label Network.

The main contributions of this paper are shown in the following three aspects: (1) This paper proposes a method for searching topological information between vertexes. According to the “three degrees of influence rule” [19] and the “six degrees of separation” [20], we only consider the vertex pairs whose path length is less than or equal to 6. For any path between $v_{x}$ and $v_{y}$, we complete the links between any two vertexes on the path. Therefore, the path length in any topological structure is less than or equal to 6, and we can store it by a graph with seven vertexes, which is called 7-subgraph; (2) This paper proposes a method based on OVCP (optimized vertex collocation profile) and supervised learning for link prediction. OVCP provides a way to convert a 7-subgraph to a unique address. For a 7-subgraph, we can calculate its address and treat it as a feature. We set a feature vector for each vertex pair $v_{x}$ and $v_{y}$. The value of a feature represents the number of occurrences of a 7-subgraph on the path. If two vertexes connect to each other in a time interval, we will set the vector of them as a true sample. Otherwise, we will set it as a false sample. Then, we use the vectors and their labels to train the supervised learning model for link prediction; (3) This paper proposes a method based on the overlapping community detection to choose vertex pairs. In this paper, we use the fast unfolding community detection algorithm and the overlapping community detection algorithm to split original networks. These two algorithms can split the network into several small communities. We assume that only the vertexes in the same community will connect to each other, which can reduce the complexity. However, the fast unfolding community detection algorithm will ignore some possible links between vertexes in different communities, while the overlapping community detection algorithm will not.

There are still some limitations to this research. (1) Most of the calculation of the method proposed in this paper focuses on the generation of feature vectors of vertex pairs, in which it is particularly time-consuming to find all paths whose path length is less than or equal to six. If the complex computational problem of finding all paths can be solved, the efficiency of this method will be greatly improved; (2) Due to the high computational complexity of algorithms and the memory limitations of a single computer, large-scale complex networks can not be effectively handled on a single machine, so a parallel environment is needed to solve this problem; (3) In our method, the weight information of vertex pairs is only used in community division, not in the ranking of vertex pairs’ scores. If the weight information of vertex pairs can be applied to the ranking, the precision of this method will be improved to a certain extent; (4) This paper only considers the undirected graph to verify the performance of our method. Extending this method to the directed graph is also involved in our future research. The fast community detection algorithm used in this paper can hold the directed graph well, while the overlapping community detection algorithm needs to extend the definition of similarity to support the directed graph. In addition, the VCP method can also hold the directed graph well, and the feature extraction can be carried out only by replacing the bit matrix with the directed matrix; (5) Our method assumes that new links are generated within communities. Although the overlapping community detection algorithm translates some links between non-overlapping communities into the communities, some links are still lost in this way. How to find out the lost links is also worth studying; (6) The prediction of unknown links in static networks is also an important topic in the problem of link prediction, which is to find the existing but undetected links in the network. How to apply this method to the prediction of unknown links will also be the direction of our research.

## Figures and Tables

**Table 1:** Symbols and description.

Symbol	Description
$\left(v_{x},v_{y}\right)$	vertex pair to be predicted
V	set of vertexes
E	set of edges
G(V,E)	graph with vertex set V and edge set E
$N_{f}$	Feature Network
$N_{l}$	Label Network
$E_{ul}$	set of unlinked edges
$E_{ue}$	set of nonexistent edges
D	adjacency matrix
ψ(G)	address of 7-subgraph
$\psi_{min}\left(G\right)$	minimal address of 7-subgraph
$k_{3,7}^{p}\left(i\right)$	union of the $p_{th}$ permutation of set {3,4,5,6,7} and set {1,2}
$VCP_{x,y}^{n}$	relation vector

**Table 2:** The details of datasets.

	Vertexes	Edges
DBLP set1	9393	22,468
DBLP set2	27,710	80,632
Facebook Friendship set1	12,715	31,882
Facebook Friendship set2	17,336	62,454
Facebook Wall set1	9787	24,232
Facebook Wall set2	11,229	29,632

**Table 3:** Time cost of each module.

Module	Time Cost
Base+OVCP+SVM	10,970 s + 20 s
Base+OVCP+NB	10,970 s + 5 s
CD+OVCP+SVM	1086 s + 9 s
CD+OVCP+NB	1086 s + 4 s
OCD+OVCP+SVM	924 s + 3 s
OCD+OVCP+NB	924 s + 2 s
VCP3	31 s
VCP4	42 s
Katz	3126 s
AA	51 s
RA	46 s
CN	48 s
Jaccard	49 s

## Data Availability

The financial event extraction dataset used in this paper is available at https://github.com/YanzuWu0311/MDPI_Entropy_LinkPrediction accessed on 1 September 2022.
